# Educational Utility of Clinical Vignettes Generated in Japanese by ChatGPT-4: Mixed Methods Study

**DOI:** 10.2196/59133

**Published:** 2024-08-13

**Authors:** Hiromizu Takahashi, Kiyoshi Shikino, Takeshi Kondo, Akira Komori, Yuji Yamada, Mizue Saita, Toshio Naito

**Affiliations:** 1 Department of General Medicine Juntendo University Faculty of Medicine Tokyo Japan; 2 Department of Community-Oriented Medical Education Chiba University Graduate School of Medicine Chiba Japan; 3 Center for Postgraduate Clinical Training and Career Development Nagoya University Hospital Aichi Japan; 4 Department of Emergency and Critical Care Medicine Tsukuba Memorial Hospital Tsukuba Japan; 5 Brookdale Department of Geriatrics and Palliative Medicine Icahn School of Medicine at Mount Sinai NY, NY United States

**Keywords:** generative AI, ChatGPT-4, medical case generation, medical education, clinical vignettes, AI, artificial intelligence, Japanese, Japan

## Abstract

**Background:**

Evaluating the accuracy and educational utility of artificial intelligence–generated medical cases, especially those produced by large language models such as ChatGPT-4 (developed by OpenAI), is crucial yet underexplored.

**Objective:**

This study aimed to assess the educational utility of ChatGPT-4–generated clinical vignettes and their applicability in educational settings.

**Methods:**

Using a convergent mixed methods design, a web-based survey was conducted from January 8 to 28, 2024, to evaluate 18 medical cases generated by ChatGPT-4 in Japanese. In the survey, 6 main question items were used to evaluate the quality of the generated clinical vignettes and their educational utility, which are information quality, information accuracy, educational usefulness, clinical match, terminology accuracy (TA), and diagnosis difficulty. Feedback was solicited from physicians specializing in general internal medicine or general medicine and experienced in medical education. Chi-square and Mann-Whitney *U* tests were performed to identify differences among cases, and linear regression was used to examine trends associated with physicians’ experience. Thematic analysis of qualitative feedback was performed to identify areas for improvement and confirm the educational utility of the cases.

**Results:**

Of the 73 invited participants, 71 (97%) responded. The respondents, primarily male (64/71, 90%), spanned a broad range of practice years (from 1976 to 2017) and represented diverse hospital sizes throughout Japan. The majority deemed the information quality (mean 0.77, 95% CI 0.75-0.79) and information accuracy (mean 0.68, 95% CI 0.65-0.71) to be satisfactory, with these responses being based on binary data. The average scores assigned were 3.55 (95% CI 3.49-3.60) for educational usefulness, 3.70 (95% CI 3.65-3.75) for clinical match, 3.49 (95% CI 3.44-3.55) for TA, and 2.34 (95% CI 2.28-2.40) for diagnosis difficulty, based on a 5-point Likert scale. Statistical analysis showed significant variability in content quality and relevance across the cases (*P*<.001 after Bonferroni correction). Participants suggested improvements in generating physical findings, using natural language, and enhancing medical TA. The thematic analysis highlighted the need for clearer documentation, clinical information consistency, content relevance, and patient-centered case presentations.

**Conclusions:**

ChatGPT-4–generated medical cases written in Japanese possess considerable potential as resources in medical education, with recognized adequacy in quality and accuracy. Nevertheless, there is a notable need for enhancements in the precision and realism of case details. This study emphasizes ChatGPT-4’s value as an adjunctive educational tool in the medical field, requiring expert oversight for optimal application.

## Introduction

The field of medical artificial intelligence (AI) has seen significant innovations, especially with the development of large language models such as ChatGPT, developed by OpenAI [1,2]. These technologies are being explored for applications across various items, including medical education [3-6], diagnostic assistance [7-9], patient health monitoring [10], and automated document creation [11,12]. However, the use of ChatGPT in health care raises serious concerns about the quality and accuracy of the information generated [13]. Accurate and reliable information is essential in health care, and inaccurate information can have harmful effects on patient health [6].

The importance of case-based learning in medicine has been well established [14]. This teaching approach is vital for medical students and health care professionals to extend their theoretical knowledge and understand the complexity and diversity of the clinical scenarios they will encounter. It fosters essential clinical reasoning and decision-making skills for accurate diagnoses and treatment plans [15,16]. Engaging with real cases helps learners develop the flexibility and adaptability needed in medical practice and encourages a more empathetic and human approach to care for patients and their families [17]. However, in teaching that involves handling actual clinical cases, creating scenarios requires a significant amount of labor, and conducting training using simulated patients can be costly, resulting in limited resources practically usable for education.

ChatGPT can easily create detailed and varied clinical scenarios that mirror actual situations, including disease types, symptom complexity, and patients’ backgrounds, without incurring high costs [18]. If it becomes clear that ChatGPT can create clinical vignettes of a level suitable for educational use, it could reduce the labor and costs for educators, allowing learners to engage with a variety of cases. Exposure to a wide range of cases allows learners to deepen their understanding of specific pathologies and treatments, strengthen their clinical judgment and problem-solving skills, and enhance their overall clinical competence, from diagnosis to treatment planning.

Moreover, in Japan, recent reforms in physicians’ work styles have mandated significant reductions in physicians’ working hours, as part of a national effort to improve work-life balance and reduce instances of overwork. These changes, although beneficial for physician well-being, have created a pressing challenge for medical education, because less time is now available for traditional in-person training and supervision. This situation underscores the urgency of using AI technologies such as generative models to efficiently supplement and enhance the training of medical professionals. However, the extent to which AI can accurately replicate clinical information and scenarios remains a critical question.

To harness the potential of AI in enhancing medical education, this study investigated the educational utility of AI-generated clinical vignettes. These clinical vignettes have the potential to be used in educational scenarios, such as simulated patient interactions, clinical reasoning, and problem-solving exercises. The integration of such AI-generated materials into medical training programs poses significant questions regarding their quality and applicability in real-world educational settings. Thus, several evaluation items were set, and a questionnaire survey of physicians specialized in general internal medicine (GIM) or general medicine (GM) was conducted, to ensure these materials meet the requisite educational standards.

First, information quality (IQ) and information accuracy (IA) were assessed to determine if the clinical vignettes adhered to fundamental quality standards, which are crucial for maintaining educational integrity. Furthermore, the metric of education usefulness (EU) was introduced to explore the actual educational value of these AI-generated clinical vignettes in medical education. Recognizing the potential disparity between AI-generated clinical vignettes and actual clinical scenarios, clinical match (CM) was evaluated to confirm the relevance and applicability of these clinical vignettes in a realistic educational framework.

Furthermore, despite the potential accuracy of the content, the precision of medical terminology and the use of the Japanese language in AI-generated cases raised concerns, necessitating a separate evaluation of terminology accuracy (TA). In addition, diagnosis difficulty (DD) was assessed to understand how variations in the complexity of presented diagnoses might affect both the accuracy of the information and its overall educational value.

This study aimed to contribute to a fundamental understanding of clinical educational content created using ChatGPT-4 by evaluating the quality and EU of clinical vignettes generated in Japanese. The objective was to determine whether the ChatGPT-4–generated vignettes effectively simulate real-life clinical scenarios, thus potentially serving as a valuable resource for medical education and training.

## Methods

### Study Design

This was an exploratory, web-based, prospective, questionnaire-based survey conducted from January 8 to 28, 2024, using a convergent mixed methods design [19].

### Selection of a Generative AI Model

ChatGPT-4 was selected for this study primarily because of its extensive use in previous medical AI research, unlike newer models such as Claude by Anthropic or Llama by Meta. This decision was driven by the availability of a robust body of literature, enabling us to directly compare our findings with well-established studies in the field.

### Medical Case Selection and Case Generation by ChatGPT-4

A total of 18 medical cases were created in Japanese using ChatGPT-4. The selection of cases was based on the 191 fundamental diseases listed in the 2022 revised *Model Core Curriculum for Medical Education* drafted by Japan’s Ministry of Education, Culture, Sports, Science, and Technology [20]. These diseases were categorized into 19 areas by organ system, and 1 disease per area was selected for this study. If an area had multiple foundational diseases, the research team chose diseases that, based on patient history and physical findings, seemed likely to suggest a diagnosis. Since 1 area (breast diseases) did not have a foundational disease listed, a total of 18 cases were included (Table 1). Each case was created through a 4-step process. Initially, the output format was set, and ChatGPT-4 was instructed to generate patient histories and physical findings based on the diagnoses. Next, whether the generated cases were typical for the diagnoses based on patient history and physical findings was verified with ChatGPT-4. The third step involved checking for the inclusion of any nonexistent information. Finally, the accuracy of the terminology used in the patient history was assessed. At no point was the generation of specific findings or histories beyond the diagnosis directed (Multimedia Appendix 1).

**Table 1:** A total of 18 cases selected from the Model Core Curriculum for Medical Education in Japan.

Case	System	Disease name
Case 1	Blood, hematopoietic, and lymphatic system	Vitamin B_12_ deficiency anemia
Case 2	Nervous system	Parkinson disease
Case 3	Skin system	Cellulitis
Case 4	Musculoskeletal system	Spinal disc herniation
Case 5	Circulatory system	Acute aortic dissection
Case 6	Respiratory system	Pulmonary thromboembolism
Case 7	Digestive system	Acute appendicitis
Case 8	Renal-urinary system	Urinary stone disease
Case 9	Reproductive system	Benign prostatic hyperplasia
Case 10	Pregnancy and childbirth	Pregnancy-induced hypertension
Case 11	Pediatrics	Febrile seizures
Case 12	Endocrine, nutritional, and metabolic system	Hyperthyroidism
Case 13	Eye and visual system	Glaucoma
Case 14	Ear, nose, throat, and oral system	Meniere disease
Case 15	Psychiatric and psychosomatic disorders	Schizophrenia
Case 16	Immune system and allergy	Systemic lupus erythematosus
Case 17	Infectious diseases	Pneumonia
Case 18	Oncology	Cervical cancer

### Study Participants

GIM or GM experts were recruited to evaluate the validity of the cases created with ChatGPT-4. Since the cases covered various specialties, the evaluators were physicians with cross-specialty knowledge in GIM or GM, all of whom had experience in medical education. The participants were recruited through mailing lists from the Japanese Society of Hospital General Medicine (JSHGM) [21], the Japan Primary Care Association (JPCA) [22], and the JHospitalist Network (JHN) [23], aiming to disseminate GIM education nationwide. Consent for participation was obtained through a Google Form.

### Questionnaire and Survey Distribution

The survey, created in Google Forms, included questions about the responding physicians’ backgrounds and questions evaluating the AI-generated cases. Background questions covered sex, year of medical license acquisition, specialty qualifications, hospital size, and work location. The evaluation of the generated cases focused on 6 aspects, which are IQ, IA, EU, CM, TA, and DD (Table 2). IQ and IA were assessed on a binary scale (yes or no), and EU, CM, TA, and DD were rated on a 5-point Likert scale (1: strongly disagree; 2: disagree; 3: neither agree nor disagree; 4: agree; 5: strongly agree). Binary responses were analyzed by converting yes to 1 and no to 0 (Multimedia Appendix 2).

**Table 2:** Contents and explanations of the 6 main questions of the questionnaire.

Item	Question	Measurement method	Scale explanation
Information quality	Do you think the medical history and physical findings provide enough quality information to recall the diagnosis?	Binary (yes or no)	Enough quality information forms the basis for accurate diagnosis process, thus answering yes or no clarifies the evaluator’s stance on the quality of information.
Information accuracy	Is the information presented in the case accurate and without contradictions?	Binary (yes or no)	Accurate information ensures reliability and effectiveness in medical education, and answering yes or no clarifies the evaluator’s stance on the accuracy of the information.
Education usefulness	Do you consider the quality of information in this case sufficient for educational purposes?	Likert scale (1-5)^a^	The usefulness of clinical vignettes in an educational context has a strong subjective element, so a Likert scale is used to capture finer impressions.
Clinical match	Does this case information reflect the medical history and physical findings you would encounter in clinical practice?	Likert scale (1-5)	Imitating realistic clinical scenarios enables learners to better prepare for situations they might face in the field. A variety of opinions and clinical experiences is important, so the Likert scale is adopted.
Terminology accuracy	Is the case information presented using appropriate medical terminology and expressions?	Likert scale (1-5)	Even if the information is accurate, the language may not be, which is why this item was included. A Likert scale is used to grade the level of language generated.
Diagnosis difficulty	How difficult do you find the diagnosis of this case?	Likert scale (1-5)	The difficulty of diagnosis serves as an indicator of the case’s complexity. A Likert scale is used to more precisely assess the level of diagnostic difficulty.

^a^1: strongly disagree; 5: strongly agree.

Respondents who rated the IQ insufficient were asked to specify reasons from among 7 options (inadequate medical history, unclear medical history, incorrect medical history, inadequate physical examination findings, unclear physical examination findings, incorrect physical examination findings, and others), allowing for multiple responses. Those who found the IA insufficient provided reasons in free-text format.

To reduce survey fatigue, the questionnaire was divided into 3 parts, each covering 6 cases, with a week allocated for each part. Responses were collected over 3 weeks, with reminders sent to nonresponders to increase response rates.

### Item-Based Data Analysis

For the 6 main items in the survey, response trends were evaluated by comparing response rates. The overall mean, SD, and 95% CI values were calculated for these rates across the 18 cases to gauge general trends and identify outliers. The calculation of mean and SD values is crucial because it helps understand the central tendency and variability of data, which supports the reliability and generalizability of the findings. All statistical analyses were performed using R (version 4.4.0; R Foundation for Statistical Computing).

### Case-Based Data Analysis

For each case, the mean and 95% CI values were calculated for responses to the 6 main items to understand case-specific response trends. Chi-square tests were conducted on binary data (IQ and IA) to evaluate whether the observed differences between groups were significant. The chi-square test was specifically chosen for its efficacy in analyzing categorical data, and it was used to determine if variations in responses were due to chance or if they reflected true differences in the medical applicability of AI-generated cases.

For items scored on a 5-point Likert scale (EU, CM, TA, and DD), which typically do not adhere to a normal distribution, the Shapiro-Wilk test was first used to confirm the nonparametric distribution of the data. Since all 4 assessed items did not follow a normal distribution, nonparametric Kruskal-Wallis tests were conducted.

In instances of significant findings, post hoc analyses were carried out using Mann-Whitney *U* tests with Bonferroni correction to adjust for multiple comparisons. This approach allowed the effective assessment of the significance of differences in perceptions across different cases, highlighting specific cases that elicited higher or lower evaluations from medical professionals.

The Mann-Whitney *U* test was used for the Likert scale items due to its appropriateness in handling data that do not meet the assumptions of normality, thus providing a more accurate measure of central tendencies across diverse case scenarios. The significant outcomes derived from these tests provide information about the consistency and variation of clinical judgments among the cases, offering critical insights into the quality of AI-generated case presentations.

### Medical Experience and Response Trends

To evaluate the relationship between medical experience and response trends, scatter plots were created, and regression lines were drawn. Linear regression analysis was conducted to determine if there was a significant association of response trends with years of medical licensure, treating the year of licensure as an independent variable and the average score for each assessment indicator as a dependent variable, calculating the slope (regression coefficient), intercept, coefficient of determination (*R* value), and *P* value.

### Qualitative Analysis

Thematic analysis of free-text responses regarding reasons for deeming IA insufficient was performed using ChatGPT-4 [24]. ChatGPT-4 processed the free-text survey results, generating a list of codes and corresponding quotations related to the research question. Themes and subthemes were then developed from these codes. Coding and theme development were validated and, if necessary, revised by 2 authors (HT and KS) using the results obtained from ChatGPT-4 [25]. The coauthors responsible for this task were physicians knowledgeable in convergent mixed methods research [26,27].

### Ethical Considerations

This study was reviewed and approved by the Juntendo University School of Medicine Research Ethics Committee, approval E23-0245, on November 10, 2023.

## Results

### Respondents

The participants were recruited through mailing lists from the JSHGM (2325 members), the JPCA (4607 members), and the JHN (3965 members), gathering 73 respondents. All 73 respondents were confirmed to be suitable. Of these, 97% (71/73) completed all surveys. The 71 participants included 64 (90%) male respondents and 7 (10%) female respondents, with licensure years ranging from 1976 to 2017. By specialty, there were 61 GIM experts, 5 GM experts, and 5 experts with both qualifications. Hospital sizes were diverse, including 35 (49%) experts from hospitals with more than 500 beds, 16 (23%) from those with 201-500 beds, 11 (15%) from those with 101-200 beds, 3 (4%) from those with fewer than 100 beds, and 6 (8%) from clinics. Respondents came from 28 (60%) of the 47 prefectures in Japan, with 1 participant from outside Japan.

### Item-Specific Questionnaires

Across the 18 cases, 76.8% (982/1278) of respondents found IQ sufficient, and 67.9% (868/1278) found IA sufficient. For the EU, 45.9% (587/1278) of respondents rated the cases as highly educational, with scores of 4. Another 15.1% (193/1278) awarded the highest score of 5. Conversely, around 19% (246/1278) expressed skepticism, giving scores of 1 or 2. CM saw a strong consensus, with over half of the participants (671/1278, 52.5%) rating the cases as highly relevant clinically, with scores of 4. Another 16% (201/1278) awarded the highest score of 5. The minority, about 13% (163/1278), gave scores of 1 or 2. TA was highly rated, with 46.2% (590/1278) of physicians expressing confidence in the accuracy of the language used (score 4), 58.7% (750/1278) expressing overall satisfaction with the TA (scores 4 and 5), and 19.6% (251/1278) providing lower scores (1 or 2). The responses to DD were more varied, since 59.5% (760/1278) of respondents found the cases relatively straightforward (scores 1 and 2), whereas higher difficulty levels (scores 4 and 5) were less frequently selected, at 13.2% (168/1278; Table 3).

Average ratings on the binary scale were 0.77 (95% CI 0.75-0.79) for IQ and 0.68 (95% CI 0.65-0.71) for IA. On the 5-point Likert scale, the averages were 3.55 (95% CI 3.49-3.60) for EU, 3.70 (95% CI 3.65-3.75) for CM, 3.49 (95% CI 3.44-3.55) for TA, and 2.34 (95% CI 2.28-2.40) for DD (Table 4).

**Table 3:** Percentage of all responses to 6 items (information quality, information accuracy, education usefulness, clinical match, terminology accuracy, and diagnosis difficulty).

Category and answer		Responses (N=1278), n (%)
**Information quality**		
	Yes	982 (76.8)
	No	297 (23.2)
**Information accuracy**		
	Yes	868 (67.9)
	No	410 (32.1)
**Education usefulness**		
	1	28 (2.2)
	2	218 (17.1)
	3	252 (19.7)
	4	587 (45.9)
	5	193 (15.1)
**Clinical match**		
	1	15 (1.2)
	2	148 (11.6)
	3	243 (19)
	4	671 (52.5)
	5	201 (15.7)
**Terminology accuracy**		
	1	32 (2.5)
	2	219 (17.1)
	3	277 (21.7)
	4	590 (46.2)
	5	160 (12.5)
**Diagnosis difficulty**		
	1	281 (22)
	2	479 (37.5)
	3	350 (27.4)
	4	139 (10.9)
	5	29 (2.3)

**Table 4:** Summary statistics of physician evaluations for AI-generated case scenarios. Information quality and information accuracy were evaluated on a binary scale of 0 or 1. Education usefulness, clinical match, terminology accuracy, and diagnosis difficulty were assessed using a Likert scale ranging from 1 to 5.

Item	Value, mean (95% CI)	Value, SD
Information quality (0 or 1)	0.77 (0.72-0.79)	0.42
Information accuracy (0 or 1)	0.68 (0.65-0.71)	0.47
Educational usefulness, Likert scale (1-5)^a^	3.55 (3.49-3.60)	1.01
Clinical match, Likert scale (1-5)	3.7 (3.65-3.75)	0.91
Terminology accuracy, Likert scale (1-5)	3.49 (3.44-3.55)	1
Diagnosis difficulty, Likert scale (1-5)	2.34 (2.28-2.40)	1.01

^a^1: strongly disagree; 5: strongly agree.

### Case-Specific Questionnaires

**Figure 1:**
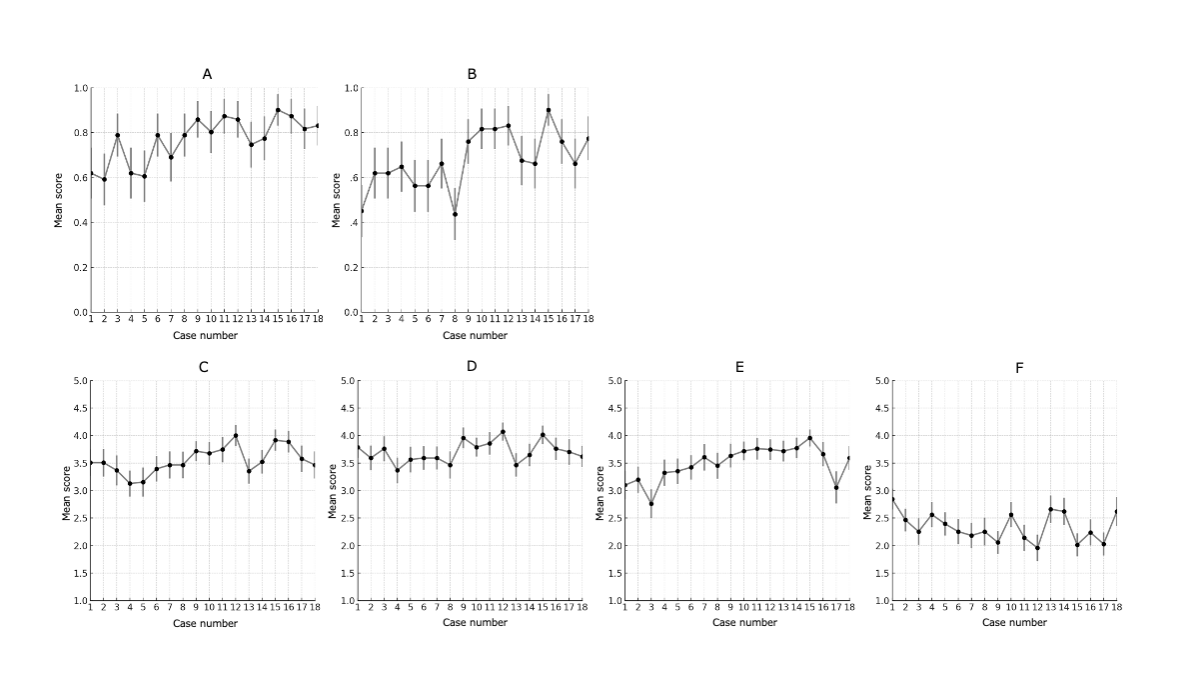
Mean and 95% CI values per case for the 6 items: (A) information quality, (B) information accuracy, (C) education usefulness, (D) clinical match, (E) terminology accuracy, and (F) diagnosis difficulty. Information quality and information accuracy were evaluated on a binary scale of 0 or 1. Education usefulness, clinical match, terminology accuracy, and diagnosis difficulty were assessed using a Likert scale ranging from 1 to 5.

Mean and 95% CI values for the 6 items (IQ, IA, EU, TA, CM, and DD) were analyzed for each of the 18 cases (Multimedia Appendix 3; Figure 1). Data analysis for each case showed significant differences in responses across all 6 items (*P*<.001 after Bonferroni correction) using chi-square tests for IQ and IA and Kruskal-Wallis tests for EU, CM, TA, and DD. Significance after correction for multiple comparisons varied, with 6 variations (6/153, 3.9% in all pairs) for IQ, 18 variations (18/153, 11.8% in all pairs) for IA, 9 variations (9/153, 5.9% in all pairs) for EU, 5 variations (5/153, 3.3% in all pairs) for CM, 22 variations (22/153, 14.4% in all pairs) for TA, and 16 variations (16/153, 10.5% in all pairs) for DD (Multimedia Appendix 4). Cases most frequently showing significant differences were case 15 (23 times), case 1 (22 times), case 12 (15 times), case 3 (13 times), and case 4 (10 times), with case 15 having the lowest *P* value combinations across all 6 main items.

Comparing the highest and lowest average scores for each item showed significant differences: (1) IQ: 0.31 between case 15 (SD 0.90) and case 2 (SD 0.59; *P*<.001); (2) IA: 0.46 between case 15 (SD 0.90) and case 8 (SD 0.44; *P*<.001); (3) EU: 0.87 between case 12 (SD 4.00) and case 4 (SD 3.13; *P*<.001); (4) CM: 0.70 between case 12 (SD 4.07) and case 4 (SD 3.37; *P*<.001); (5) TA: 1.20 between case 15 (SD 3.96) and case 3 (SD 2.76; *P*<.001); and (6) DD: 0.89 between case 1 (SD 2.85) and case 12 (SD 1.96; *P*<.001).

### Reasons for Insufficient IQ

Overall, 23.2% (297/1278) of respondents who scored the IQ insufficient cited incorrect patient history (23/1278, 1.8% of all cases) and incorrect physical findings (19/1278, 1.5% of all cases). Other responses indicated that, although AI-generated cases were useful for generating patient histories, improvements were needed in generating physical findings, the naturalness of language, and the accuracy of medical terminology (Multimedia Appendix 5).

### Reasons for Insufficient IA

In exploring the reasons for inadequate IA as reported by respondents (410/1278, 32.1%), thematic analysis of open-ended responses identified 5 primary themes, which are (1) documentation clarity and precision, (2) consistency and reliability of clinical information, (3) appropriateness and contextual relevance, (4) comprehensiveness of diagnostic and treatment insights, and (5) patient-centered reporting (Table 5).

**Table 5:** Results of thematic analysis of the reasons for respondents’ answers that information is insufficiently accurate.

Themes and subthemes		Quotes
**Documentation clarity and precision**		
	Detail and specificity	I would like to know about gastrectomy.
	Appropriate terminology	Do not use the expression “mesh-like sensation.”
**Consistency and reliability of clinical information**		
	Avoiding contradictions	Patient had a checkup at a nearby clinic, and no abnormalities were found. The details of the examination are unknown, but it is likely that a blood count was performed even if vitamins were not measured. Given that iron supplements were prescribed, it can be inferred that anemia was observed in the blood test. The statement “no abnormalities were found” is contradictory.
	Ensuring accuracy in descriptions	The description “oral cavity: erythema of the tongue, normal dental health” is hard to understand.
**Appropriateness and contextual relevance**		
	Contextual relevance to the patient’s condition	It mentions obstetric history despite being about a male.
	Practicality in clinical settings	I think the case itself is typical, but it seems unlikely that there would be time to conduct such a detailed physical examination on a patient experiencing severe chest pain accompanied by shortness of breath and cold sweats, and who has abnormally high blood pressure.
**Comprehensiveness in diagnosis and treatment insights**		
	Diagnostic clarity	The name of the prescribed antibiotic is needed.
	Logical treatment choices	It says an antianxiety medication was prescribed, but it is clearly a case of auditory hallucinations. It is unlikely any doctor would prescribe just an antianxiety medication in such a situation.
**Patient-centered reporting**		
	Incorporating patient history and experience	It is strange to get a pneumococcal vaccine at 60 with only a history of high blood pressure.
	Detailed symptom documentation	The main complaint is fatigue, but the details of the fatigue (changes in ADL^a^, IADL^b^, etc) are not described.

^a^ADL: activities of daily living.

^b^IADL: instrumental activities of daily living.

#### Documentation Clarity and Precision

Concerning documentation clarity and precision, issues were highlighted regarding the vagueness of specific information, such as details of surgeries and explanations of adjunct treatments. It was also noted that consistent use of medical terminology is demanded, with unclear or incorrect use of specialized terms leading to misunderstanding of information.

#### Consistency and Reliability of Clinical Information

For the consistency and reliability of clinical information, reported instances raised doubts about the trustworthiness of information, including contradictions in clinical findings and discrepancies in physical examinations. Medical documents should contain information relevant to the specific situations or conditions of patients, yet instances of unnecessary or irrelevant information were observed.

#### Appropriateness and Contextual Relevance

Concerns about appropriateness and contextual relevance were particularly noted in examples, such as the practicality of clinical tests in emergencies and the inclusion of information unrelated to patients’ medical histories.

#### Comprehensiveness of Diagnostic and Treatment Insights

In diagnostic and treatment insights, comprehensive and detailed information is required. However, instances were observed where descriptions of specific medications were lacking or the rationale for treatment choices was questioned. It was pointed out that, in AI-generated case scenarios, detailed clinical data and clear justifications for treatment choices are crucial. Comprehensive documentation of patients’ histories and experiences is essential for delivering patient-centered care, yet deficiencies were noted in the detailed reporting of specific symptoms or the consistency of patients’ actions and histories, indicating insufficient patient-centered perspectives in reporting.

### Medical Experience and Response Trends

**Figure 2:**
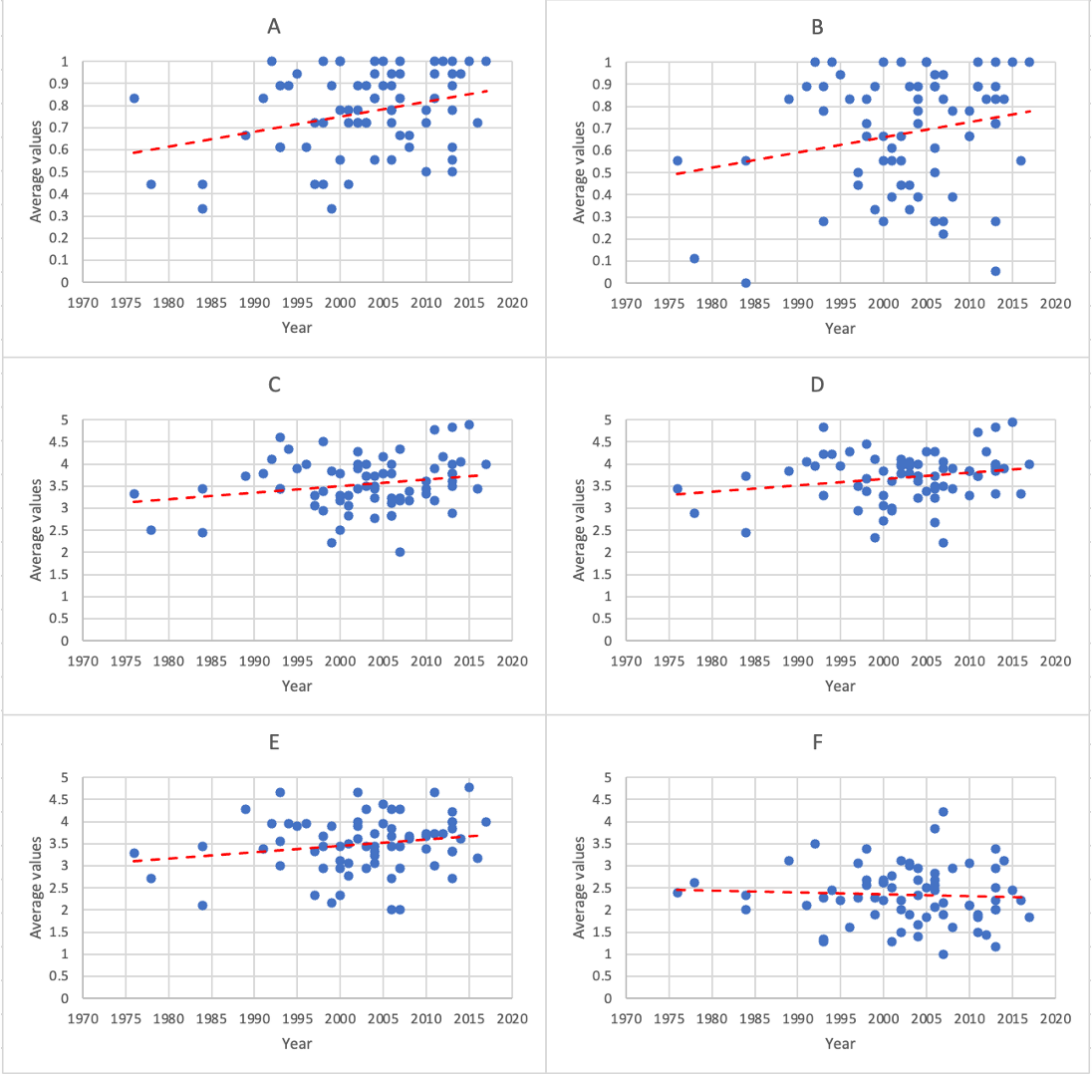
Scatter plot with regression lines showing the relationship between years since obtaining the medical license and average values for the 6 evaluated items: (A) information quality, (B) information accuracy, (C) education usefulness, (D) clinical match, (E) terminology accuracy, and (F) diagnosis difficulty.

In the scatter plots and regression lines of the years since obtaining a medical license, the 6 main items, IQ, IA, EU, CM, and TA, were all rated lower by physicians with longer careers and higher by those with shorter careers. DD, although mostly horizontal, was slightly inclined downward, indicating a trend where physicians with longer experience rated it more difficult, and those with shorter experience rated it easier (Figure 2). Linear regression analysis showed a significant association for IQ (*P*=.01). IA (*P*=.06), EU (*P*=.07), CM (*P*=.06), and TA (*P*=.10) did not show significant associations, although the *P* values were low. The difficulty of diagnosis (*P*=.62) showed no relationship with the length of medical experience (Multimedia Appendix 6).

## Discussion

### Principal Findings

In this study, Japanese medical cases generated by ChatGPT-4 were evaluated by GIM or GM experts. A high response rate (71/73, 97%) and a diverse participant demographic in terms of years of medical licensure, hospital size, and geographical location of affiliated organizations support the reliability of current findings. Evaluations across 6 key items (IQ, IA, EU, CM, TA, and DD) indicated that AI-generated medical cases possess a certain level of quality and accuracy suitable for use as clinical educational materials.

Overall, 76.8% (982/1278) of GIM or GM experts rated the IQ of the AI-generated cases as adequate. A very small percentage of cases were noted for having clear errors in medical history (23/1278, 1.8%) and physical examination findings (19/1278, 1.5%), with the total percentage of cases with clear errors in either item being only 3.3% (42/1278). This suggests that, despite some mistakes and lack of information, the majority of specialists found no significant issues with the quality of the generated cases, indicating that ChatGPT-4’s case generation likely has fundamental reliability and accuracy. Similar to this study, research using ChatGPT-4 to generate 202 clinical vignettes in Japanese also involved evaluation by three physicians for medical and linguistic accuracy. It was found that 97% (196/202) of these clinical vignettes required some modifications to be deemed usable, supporting these findings [28].

The 5-point Likert scale used rates “1” as the lowest and “5” as the highest evaluation, with “3” representing a neutral or undecided assessment. Each item rated as “4” suggests effectiveness. The scores for EU (3.55), CM (3.70), and TA (3.49) were between 3 and 4, indicating a level requiring modifications for practical educational use. In addition, the score for DD ranged between 2 and 3, suggesting it was easier than average. This implies that, with appropriate modifications, even relatively simple clinical vignettes could be effectively used for educational purposes.

The analysis of responses scored as insufficient IA (410/1278, 32.1%) showed that the AI-generated cases sometimes failed to provide medical information deemed necessary by GIM or GM experts, lacking specific information depending on the disease or not aligning with what the GIM or GM experts considered relevant clinical information. This suggests that, although ChatGPT-4 can generate disease information to some extent, it may not accurately represent actual clinical scenarios. Furthermore, instances of inappropriate use of Japanese language and expressions were also pointed out, highlighting the need for verification of the appropriateness and accuracy of medical information, representation of clinical scenarios, and use of Japanese language when using ChatGPT-4–generated cases for educational purposes.

The analysis of the responses to the 18 cases across the 6 key items showed significant variance through multigroup chi-square and Kruskal-Wallis tests. Specifically, case 15 was included in the combination that showed significance for all 6 items but was rated the third easiest in terms of diagnostic difficulty, and it ranked in the top 2 for the other 5 items, indicating a high evaluation. This suggests that case 15, a psychiatric case, was recognized from the medical history as a psychiatric disorder, and physical examination findings were not involved in the diagnosis. Among the reasons for inadequate IA ratings, the responses that the cases produced by ChatGPT-4 indicated that the history was accurate, but that the physical examination findings remained a challenge, supporting the reason why case 15 received a high rating.

Comparing cases with the highest and lowest mean values across all 6 items, not only were there significant differences across all items, but there were also substantial differences between the highest and lowest values. For instance, there was a 0.46 difference in the accuracy of information between case 15 and case 8, representing a significant difference, with 33 (46%) out of 71 respondents answering “yes.” Similarly, a significant difference of 1.20 was observed in TA between case 15 and case 3. These results suggest that, although AI-generated cases generally maintained a certain level of accuracy, there was significant variability in quality across cases for the 6 key items.

Analysis of scatter plots and regression lines of the relationship between years of medical licensure and response trends per case suggested a potential correlation between the length of medical practice and response tendencies. Not only IQ, which was significant on linear regression analysis, but also IA, EU, CM, and TA had low *P* values, suggesting that longer-practicing physicians developed more stringent criteria over time due to their increased knowledge and experience. It might also indicate that less experienced physicians are more receptive to new technologies and tools, valuing the utility of AI-generated cases more highly.

### Limitations

This study focused on 18 cases of basic diseases and did not evaluate the maintenance of IQ and accuracy in complex cases. In addition, the evaluations were conducted by GIM or GM experts without obtaining assessments from specialists in various fields. Actual interaction and testing with learners are necessary to assess the usefulness of teaching clinical vignettes, but no interaction or testing with learners was conducted in this study. It is also important to note that this study was based on the use of ChatGPT-4 and that different outcomes might have been observed with other AI models, such as Claude by Anthropic or Llama by Meta. The evaluation structure was designed to ensure a comprehensive assessment of the AI-generated clinical vignettes. However, the absence of clear evaluative standards for respondents remains a limitation, potentially leading to variability in their interpretations and affecting the validity of the findings. The proportion of female respondents in this study was 9.5% (7/71). According to the 2020 data from the Ministry of Health, Labour, and Welfare, female physicians make up 22.8% (77,546/339,623) of all physicians in Japan, indicating a disproportionately higher number of male respondents in this study [29]. Finally, the questionnaire used was newly created and did not undergo a pilot test.

In this study, it was suggested that when creating clinical vignettes using the current ChatGPT-4, user corrections are necessary. Given the potential risks associated with the long-term use of AI, such as the homogenization of medical knowledge and the perpetuation of errors present in the training data, implementing this approach may be crucial in mitigating these risks.

### Conclusions

This study showed that, although ChatGPT-4–generated medical cases contain minor mistakes, the likelihood of significant errors is low, and they possess a certain level of quality and accuracy of information. However, when evaluating individual cases, there is considerable variability in accuracy, underscoring the need for verification of the provision of appropriate medical information, representation of clinical scenarios, and accuracy of the Japanese language when using these AI-generated cases for educational purposes.

## Appendix

Multimedia Appendix 1 Prompts used for generating clinical vignettes with ChatGPT-4.

Multimedia Appendix 2 Questionnaire form for case 1.

Multimedia Appendix 3 Physicians’ assessments of artificial intelligence–generated cases, with mean (95% CI) values. Information quality and information accuracy were evaluated on a binary scale of 0 or 1. Education usefulness, clinical match, terminology accuracy, and diagnosis difficulty were assessed using a Likert scale ranging from 1 to 5.

Multimedia Appendix 4 Results of chi-square tests for information quality and information accuracy and of Kruskal-Wallis tests for education usefulness, clinical match, terminology accuracy, and diagnosis difficulty.

Multimedia Appendix 5 Summary of reasons for deeming the information quality insufficient.

Multimedia Appendix 6 Results of linear regression analysis.

## Abbreviations

AI: artificial intelligence
CM: clinical match
DD: diagnosis difficulty
EU: education usefulness
GIM: general internal medicine
GM: general medicine
IA: information accuracy
IQ: information quality
JHN: JHospitalist Network
JPCA: Japan Primary Care Association
JSHGM: Japanese Society of Hospital General Medicine
TA: terminology accuracy
