# Supplementary material for: Educational Utility of Clinical Vignettes Generated in Japanese by ChatGPT-4: Mixed Methods Study
Source: JMIR Med Educ. 2024 Aug 13;10:e59133. doi: 10.2196/59133 (PMC11350316; doi:10.2196/59133)
Supplement: Multimedia Appendix 1 [file mededu_v10i1e59133_app1.docx]

*1^st^ Prompt for Patient History:*

You are a physician, about to review a patient’s information for a case study. Please present the patient information step by step for a patient who has been diagnosed with “diagnosis name.” at their first visit. The patient history should include the following:

Patient History:

- Basic Information: Age, sex, nationality.
- Chief Complaint: The main reason or specific symptoms that led the patient to seek medical attention. This symptom should be the most severe or the direct trigger for seeking medical care.
- Current Illness History (200-600 characters): Describe the type of discomfort (what the patient is struggling with) and all information necessary for differential diagnosis in a chronological order. Include both positive and negative findings relevant to the differential diagnosis, even if negative (e.g., “no fever”). For pain as the main complaint, detail the OPQRST aspects and include triggers and relieving factors. Also include any visits to other medical facilities, with dates, diagnoses, treatments, and how treatments affected the condition.
- Past Medical History: Past illnesses, surgeries, injuries, hospitalizations (include events related to the current chief complaint in the current illness history). Detail chronic diseases, serious medical conditions, specific diagnoses, types of surgeries, severity of injuries, and treatment progressions. For women, include obstetric history.
- Medication History: Current or past medications, supplements, herbal remedies. Include side effects, dosages, and duration. Detail any changes in response or effects to specific drugs or supplements.
- Allergy History: Known allergies to drugs, food, or the environment. Include specific substances, reaction severity, and if applicable, carry information like EpiPens.
- Family History: Hereditary diseases, causes of death at a young age, etc. Detail diseases in direct relatives (parents, siblings, children), specific diagnoses, family members’ health status, and potential for heredity.
- Lifestyle: Smoking history (amount, duration), alcohol intake (frequency, quantity), sleep, diet (particularly regularity and nutritional status), exercise (frequency, type), sexual activity (use of protection, number of partners). Include how these habits affect health. For women, also detail menstrual history.
- Social History: Job (type, work environment), family structure, home environment (stress level, living situation), pets (type, frequency of contact). Consider potential health impacts of the living environment.
- Travel History: Countries or regions visited, duration of stay, activities. Include risks of infectious diseases and preventive measures taken.
- Vaccination History: Specific types and dates of vaccinations, number of doses. Provide information on immunity status or resistance to specific infectious diseases.
- Mental State: Signs of emotional stability, stress, anxiety, depression. Include past mental disorders and current treatments (counseling, medication, etc.).

**Note for Creation:**

- Answer in words mainly for sections other than the current illness history.
- Ensure the history is typical for the diagnosis.
- Confirm that the history is characteristic for the given diagnosis and does not contain the diagnosis term.

**Confirmation**:

- Ensure the patient history is specific to the diagnosis.
- Check that the diagnosis term is not included in the history.

*2^nd^ Prompt for Patient History* (Repeat until no revisions are necessary):

Verify step by step if this patient history is typical for the “diagnosis name.” If there are any areas for improvement, please inform me.

*3^rd^* *Prompt for Patient History* (Repeat until no revisions are necessary):

Step by step, confirm that this patient history does not include any information that does not actually exist.

4^th^ *Prompt for Patient History* (Repeat until no revisions are necessary):

Step by step, check if the word choices in this patient history are accurate.

*1^st^ Prompt for Physical Examination:*

List all physical findings characteristic of “diagnosis name” and describe the patient’s physical examination findings step by step. The physical examination should include:

1. Basic Information: Height, weight, BMI.
2. Vital Signs: Level of consciousness (GCS), blood pressure (right arm, left arm, standing, sitting), heart rate (HR, pulse regularity), respiratory rate (RR, breathing pattern), oxygen saturation (SpO_2_, oxygen inhalation concentration), body temperature (skin temperature).
3. Head & Facial: Appearance, hair, scalp, skull, skin condition (pigmentation, moisture, rash, scars), eyelid conjunctiva, eyeball conjunctiva, vision, pupil reaction (to light, symmetry), field of vision test, facial nerve evaluation.
4. Ears, Nose, Mouth, Throat: Ear inspection and auscultation (discharge, wax, eardrum condition), nose inspection (nasal cavity, septum), oral cavity inspection (teeth health, mucosa, tongue movement).
5. Neck: Lymph node enlargement, thyroid palpation (size, hardness, nodules), vascular inspection and auscultation (presence of bruits, pulse strength), cervical spine mobility, neck posture, skin condition.
6. Chest: Inspection (shape, respiratory movements, breast condition), auscultation (breath sounds, heart sounds, rubs, murmurs), percussion, palpation (fremitus, tactile vocal fremitus, size).
7. Abdomen: Inspection (abdominal distension, rash, ecchymosis, pregnancy), auscultation (bowel sounds, vascular murmurs, quality of murmurs), percussion (liver, spleen size), palpation (abdominal hardness, pain location, rebound tenderness, guarding, masses).
8. Limbs: Inspection (rash, edema, nail condition), palpation (swelling, warmth, pain, arterial pulsation), joint mobility (contracture, joint pain), circulatory and neurological evaluation (peripheral circulation, sensation, muscle strength, reflexes), posture, gait, muscle atrophy or hypertrophy.
9. Nervous System: Cranial nerve functions (vision, hearing, smell, taste, touch, motor functions, pain, temperature sense, proprioception), reflexes (tendon reflexes, abnormal reflexes, cutaneous reflexes), cognitive function tests (MMSE or MOCA), sitting and standing balance, coordination movements, finger-nose coordination.

**Note for Creation**: Physical examination does not include echocardiography. Ensure the physical findings are most typical for the diagnosis and do not list the disease name.

**Confirmation**: Ensure all physical findings characteristic of the diagnosis are covered and the diagnosis term is not included in the physical findings.

*2^nd^ Prompt for Physical Examination* (Repeat until no revisions are necessary):

Verify step by step if these physical findings are typical for the “diagnosis name” and suggest improvements if any.

*3^rd^ Prompt for Physical Examination* (Repeat until no revisions are necessary):

Confirm step by step that the physical findings do not include any nonexistent information.

*4^th^ Prompt for Physical Examination* (Repeat until no revisions are necessary):

Check step by step if the wording of the physical findings is general.
