# Supplementary material for: Educational Utility of Clinical Vignettes Generated in Japanese by ChatGPT-4: Mixed Methods Study
Source: JMIR Med Educ. 2024 Aug 13;10:e59133. doi: 10.2196/59133 (PMC11350316; doi:10.2196/59133)
Supplement: Multimedia Appendix 2 [file mededu_v10i1e59133_app2.docx]

Title: Exploratory Evaluation of Case Generation by ChatGPT

1. Research Background

In recent years, the evolution of artificial intelligence (AI) has introduced a new technology known as generative AI. This generative AI, leveraging natural language processing, has the capability to produce documents that seem as though they were written by humans, leading to anticipated applications across various industries. In the medical field, there are ongoing efforts to use this generative AI to create medical case reports. However, it is known that generative AI has the tendency to “lie,” necessitating verification of the medical accuracy of the generated cases.

1. Research Objective

The primary objective of this study is to evaluate the quality of information in medical cases created by generative AI. Specifically, we aim to verify the medical history and physical findings of cases generated by AI and evaluate the following aspects:

- Whether the presented cases are medically accurate.
- The extent to which the cases generated by AI contain “lies.”
- Whether the cases are suitable for educational purposes.
- Whether the cases contain realistic information.
- Whether the cases are expressed in an understandable manner.

This will explore the reliability of cases provided by generative AI and their potential use in medical education.

1. Research Method

3.1 Selection of Cases for Evaluation In this study, medical cases generated by AI will be evaluated. These cases are based on 18 diseases listed in the “Medical Education Model Core Curriculum (Revised Edition, 2022)” and will include one disease from each category, totaling 18 cases prepared by the research team based on medical history and physical findings.

3.2 Evaluation Method Participants will review each provided case and evaluate the following aspects:

- Medical accuracy of the case
- Suitability of the case for educational purposes
- Whether the case contains realistic information
- Whether the case is expressed in an understandable manner

3.3 Evaluation Time The evaluation of each case is expected to take approximately 40-60 minutes. The questionnaire can be saved and resumed as needed.

1. Participation Conditions

This study requires participants to have knowledge and experience in interpreting and analyzing cases, therefore, being a general practitioner or an internal medicine specialist is required.

1. Role of Participants

Participants will review a series of cases generated by AI and evaluate whether the medical history and physical findings provide medically accurate information, are suitable for educational purposes, contain realistic information, and are expressed in an understandable manner.

1. Data Management and Privacy

Information collected in this study will be used solely for achieving our research objectives. We may consider conducting interviews to seek detailed opinions or information from specific respondents, participation in which will be voluntary. All data, except for interviews, will be strictly managed by our research team and anonymized during analysis and reporting. No personal identification will be possible.

1. Risks and Benefits

There are no direct risks associated with this study. The information provided by participants will contribute to improving the method of providing medical case information and to the overall development of medical education.

1. Freedom to Participate and Withdraw

Participation in this study is voluntary. Participants can withdraw at any time without any penalty. Non-participation or withdrawal will not affect participants’ current or future professional standing or evaluations. To withdraw consent, please send an email to the study representative expressing your intention to withdraw.

1. Conflict of Interest Statement

The researchers and the research team involved in this study have not received, and will not receive, any financial or non-financial benefits related to this study. Thus, our judgment of the study results is unbiased. We declare that there is no improper influence or interference from external third parties or institutions on the purpose, methods, interpretation, and publication of the study results. The researchers and research team will strive to avoid conflicts of interest throughout the study to maintain transparency and fairness.

1. Contact Information for Research Representatives

This study is conducted with the permission of Professor Toshio Naito, Head of the Department of General Medicine, Juntendo University School of Medicine. For inquiries or clarifications about this study, please contact Associate Professor Hiromizu Takahashi of the Department of General Medicine, Juntendo University School of Medicine (hrtakaha@juntendo.ac.jp).

Email Address: ________________________

Please provide your affiliation: _______________________

Please provide your name: ________________________

Case 1

*Medical History*

[Basic Information] Age: 60 years old, Sex: Female, Nationality: Japanese

[Chief Complaint] Fatigue

[History of Present Illness] Began feeling fatigued about a year ago, recently started experiencing numbness in the legs during walking and trembling hands, along with decreased concentration and memory. Fatigue is especially strong during the day, with an increased frequency of naps. Often told by family that “you look pale,” and sometimes feels tongue pain. Tested at a local clinic but no abnormalities were found, and prescribed iron supplements that did not improve symptoms.

[Past Medical History] Gastric ulcer (cured 20 years ago), Gastrectomy (20 years ago), Births: 2

[Medication History] Iron supplements (for 3 months, no improvement in symptoms), Multivitamin supplement (taken regularly)

[Allergy History] None

[Family History] Mother: Diabetes, Father: Stroke (cause of death), Sister: Healthy

[Lifestyle] Smoking history: None, Drinking history: Once a week, one drink, Sleep: 6 hours/day, Diet: Regular, but low intake of meat and fish, Exercise: Walking twice a week, Menstrual history: None (postmenopausal)

[Social History] Occupation: Office work, Family composition: Husband, two adult children, Pets: One cat

[Travel History] None

[Vaccination History] Influenza vaccine (annually), Pneumococcal vaccine (5 years ago)

[Mental State] Emotional stability: Good, Stress: Work-related busyness, Signs of anxiety/depression: None

*Physical Findings*

[Basic Information] Height: 157 cm, Weight: 52 kg, BMI: 21.1 kg/m^2^

[Vital Signs] Consciousness level: GCS 15/15, Blood pressure: Right arm 120/80mmHg, Left arm 122/81mmHg, Standing 115/78mmHg, Seated 118/79mmHg, Heart rate: HR 82 bpm, regular, Respiratory rate: RR 18/min, normal respiratory pattern, Oxygen saturation: SpO_2_ 98% (room air), Body temperature: 36.7 C

[Head and Face] Facial appearance: Pale, Hair: Normal, Scalp: Normal, Skin condition: Uniform color, Eyelid conjunctiva: Anemia signs, Eyeball conjunctiva: Normal, Vision: Normal, Pupil reaction: Normal light reaction, symmetrical, Visual field test: Normal, Facial nerve: Normal

[Ears, Nose, Mouth, Throat] Ears: Normal, Nose: Normal, Oral cavity: Tongue erythema, healthy teeth

[Neck] Neck lymph nodes: None, Thyroid: Normal, Vessels: Normal, Neck vertebrae mobility: Normal, Skin condition: Normal

[Chest] Inspection: Normal, Auscultation: Clear breath sounds, rhythmic heart sounds without murmurs, Percussion: Normal, Palpation: Normal

[Abdomen] Inspection: Flat, Auscultation: Normal bowel sounds, Percussion: Normal liver and spleen, Palpation: Soft abdomen, no rebound or guarding.

[Extremities] Inspection: Normal, Palpation: Normal, Joint mobility: Normal, Circulatory and nervous systems: Normal

[Nervous System] Cranial nerve function: Normal, Sensory evaluation: Slight decrease in peripheral sensation in the lower limbs, especially in reticular sensation and touch, Motor function: Mild hand tremor, overall muscle strength within normal range, Reflexes: Normal knee and ankle reflexes, Babinski reflex negative, Cognitive function test: MMSE 28/30, Posture and balance: Mild instability while standing, Hand coordination: Normal

Q1: The diagnosis for this case is “Vitamin B12 Deficiency.” Do you think the medical history and physical findings provide enough quality information to recall the diagnosis?

Yes or No

Q2: For those who selected No, please provide the reason(s) (multiple selections possible):

- Insufficient information in the medical history
- Unclear information in the medical history
- Obvious mistakes in the medical history
- Insufficient information in the physical findings
- Unclear information in the physical findings
- Obvious mistakes in the physical findings
- Other...

Q3: Is the information presented in the case accurate and without contradictions?

Yes or No

Q4: For those who answered No, please describe all inaccuracies or contradictions.

Q5: Do you consider the quality of information in this case sufficient for educational purposes?

1. Strongly Disagree - 5. Strongly Agree

Q6: Does this case information reflect the medical history and physical findings you would encounter in clinical practice?

1. Strongly Disagree - 5. Strongly Agree

Q7: Is the case information presented using appropriate medical terminology and expressions?

1. Strongly Disagree - 5. Strongly Agree

Q8: How difficult do you find the diagnosis of this case?

1. Very Easy - 5. Very Difficult

Q9: Please share any additional observations you have about this case.
