# Supplementary material for: Educational Utility of Clinical Vignettes Generated in Japanese by ChatGPT-4: Mixed Methods Study
Source: JMIR Med Educ. 2024 Aug 13;10:e59133. doi: 10.2196/59133 (PMC11350316; doi:10.2196/59133)
Supplement: Multimedia Appendix 4 [file mededu_v10i1e59133_app4.docx]

**Multimedia Appendix 4.**

|  | p<0.05 | | p<0.01 | | p<0.001 | |
| --- | --- | --- | --- | --- | --- | --- |
| Information Quality | Case 1 | Case 15 | Case 2 | Case 15 |  |  |
|  | Case 2 | Case 11 |  |  |  |  |
|  | Case 2 | Case 16 |  |  |  |  |
|  | Case 4 | Case 15 |  |  |  |  |
|  | Case 5 | Case 15 |  |  |  |  |
| Information Accuracy | Case 1 | Case 18 | Case 1 | Case 11 | Case 1 | Case 15 |
|  | Case 1 | Case 16 | Case 1 | Case 10 | Case 1 | Case 12 |
|  | Case 1 | Case 9 | Case 5 | Case 15 | Case 8 | Case 15 |
|  | Case 2 | Case 15 | Case 6 | Case 15 | Case 8 | Case 12 |
|  | Case 3 | Case 15 |  |  | Case 8 | Case 11 |
|  | Case 8 | Case 18 |  |  | Case 8 | Case 10 |
|  | Case 8 | Case 16 |  |  |  |  |
|  | Case 8 | Case 9 |  |  |  |  |
| Education Usefulness | Case 4 | Case 11 | Case 5 | Case 15 | Case 4 | Case 12 |
|  | Case 6 | Case 12 | Case 5 | Case 16 | Case 4 | Case 15 |
|  |  |  | Case 12 | Case 13 | Case 4 | Case 16 |
|  |  |  |  |  | Case 5 | Case 12 |
| Clinical Match | Case 4 | Case 9 | Case 4 | Case 12 |  |  |
|  | Case 13 | Case 15 | Case 4 | Case 15 |  |  |
|  |  |  | Case 12 | Case 13 |  |  |
| Terminology Accuracy | Case 1 | Case 14 | Case 3 | Case 7 | Case 1 | Case 15 |
|  | Case 1 | Case 11 | Case 3 | Case 18 | Case 2 | Case 15 |
|  | Case 1 | Case 12 | Case 15 | Case 17 | Case 3 | Case 9 |
|  | Case 2 | Case 14 |  |  | Case 3 | Case 10 |
|  | Case 2 | Case 11 |  |  | Case 3 | Case 11 |
|  | Case 3 | Case 8 |  |  | Case 3 | Case 12 |
|  | Case 4 | Case 15 |  |  | Case 3 | Case 13 |
|  | Case 5 | Case 15 |  |  | Case 3 | Case 14 |
|  | Case 6 | Case 15 |  |  | Case 3 | Case 15 |
|  |  |  |  |  | Case 3 | Case 16 |
| Diagnosis Difficulty | Case 1 | Case 6 | Case 1 | Case 3 | Case 1 | Case 9 |
|  | Case 1 | Case 8 | Case 1 | Case 7 | Case 1 | Case 12 |
|  | Case 1 | Case 16 | Case 1 | Case 11 | Case 1 | Case 15 |
|  | Case 4 | Case 12 |  |  | Case 1 | Case 17 |
|  | Case 10 | Case 12 |  |  |  |  |
|  | Case 12 | Case 13 |  |  |  |  |
|  | Case 12 | Case 14 |  |  |  |  |
|  | Case 13 | Case 15 |  |  |  |  |
|  | Case 13 | Case 17 |  |  |  |  |
